# Sedentary behaviors and risk of depression: a meta-analysis of prospective studies

**DOI:** 10.1038/s41398-020-0715-z

**Published:** 2020-01-22

**Authors:** Yuchai Huang, Liqing Li, Yong Gan, Chao Wang, Heng Jiang, Shiyi Cao, Zuxun Lu

**Affiliations:** 1 School of Public Health, Tongji Medical College, Huazhong University of Science and Technology, Wuhan, Hubei China; 2 Department of Management Science and Engineering, School of Economics and Management, Jiangxi Science and Technology Normal University, Nanchang, Jiangxi China; 3 Centre for Alcohol Policy Research, School of Psychology and Public Health, La Trobe University, Melbourne, VIC Australia; 4 Centre for Health Equity, Melbourne School of Population and Global Health, The University of Melbourne, Melbourne, VIC Australia

**Keywords:** Scientific community, Depression

## Abstract

Epidemiological evidence on the association between sedentary behaviors and the risk of depression is inconsistent. We conducted a meta-analysis of prospective studies to identify the impact of sedentary behaviors on the risk of depression. We systematically searched in the PubMed and Embase databases to June 2019 for prospective cohort studies investigating sedentary behaviors in relation to the risk of depression. The pooled relative risks (RRs) and 95% confidence intervals (CIs) were calculated with random-effect meta-analysis. In addition, meta-regression analyses, subgroup analyses, and sensitivity analyses were performed to explore the potential sources of heterogeneity. Twelve prospective studies involving 128,553 participants were identified. A significantly positive association between sedentary behavior and the risk of depression was observed (RR = 1.10, 95% CI 1.03–1.19, *I*^2^ = 60.6%, *P* < 0.01). Subgroup analyses revealed that watching television was positively associated with the risk of depression (RR = 1.18, 95% CI 1.07–1.30), whereas using a computer was not (RR = 0.99, 95% CI 0.79–1.23). Mentally passive sedentary behaviors could increase the risk of depression (RR = 1.17, 95% CI 1.08–1.27), whereas the effect of mentally active sedentary behaviors were non-significant (RR = 0.98, 95% CI 0.83–1.15). Sedentary behaviors were positively related to depression defined by clinical diagnosis (RR = 1.08, 95% CI 1.03, 1.14), whereas the associations were statistically non-significant when depression was evaluated by the CES-D and the Prime-MD screening. The present study suggests that mentally passive sedentary behaviors, such as watching television, could increase the risk of depression. Interventions that reduce mentally passive sedentary behaviors may prevent depression.

## Introduction

Sitting is a typical way to relax and get efficient storage of energy in a short time^1^. As a common type of sitting behavior, sedentariness refers to any sitting behavior with an energy expenditure ≤1.5 metabolic equivalents^2^. In fact, sedentary behaviors are very common in daily life, especially for many occupations, such as researchers, clerks, drivers, and programmers. There is currently no clear definition of mentally passive and mentally active sedentary behaviors. In general, behaviors including watching television, sitting around, listening, and talking while sitting are considered as “mentally passive”, whereas using a computer, reading books or newspapers, car driving, attending a meeting, knitting, or sewing are interpreted as “mentally active”^3,4^. Studies have found that, sedentary behaviors could have adverse impacts on physical health, increasing the risk of chronic diseases including cancer, cardiovascular diseases, diabetes mellitus, and so on^5–10^. Mental health damage such as stress, dementia, and sleeping problem caused by sedentary behaviors have also been reported in a number of studies^11–14^. In recent years, emerging studies have found different effects between mentally active and mentally passive sedentary behaviors on an individual’s well-being. A cross-sectional study found that mentally passive sedentary behaviors might be deleterious, and mentally active sedentary behaviors could be beneficial to health^3^. Another 13-year prospective study found that substituting mentally passive with mentally active sedentary behaviors may reduce the risk of depression^4^. As sedentary behavior is so common in our daily life, more attention should be paid to its effects on health, and those of specific mentally passive and mentally active sedentary behaviors if necessary.

Depression is a common mental disorder and major human blight that affects up to 25% of women and 12% of men^15,16^. According to the World Health Organization, ~ 350 million people had suffered from depression in 2010^16^. Specific clinical and therapeutic features for depression have been described in childhood and the elderly respectively^17,18^. Research has found depression to be more common in women than men, and sex differences have been found to have age specific effects on the risk of depression^19–22^. A previous meta-analysis, that pooled the results of cohort and case–control studies, suggested that there was a positive relation between sedentary behaviors and the risk of depression for the entire population^23^. Another meta-analysis published in 2016 concluded that sedentary behaviors were associated with depression in a non-linear relation for adolescents^24^. However, it remains controversial whether this effect was consistent in adults, as a great deal of research have contradicted their findings or drawn out statistically non-significant conclusions. A prospective study with a follow-up of 9.3 years indicated that sedentary behaviors increased the risk of depression^25^. Although another prospective cohort study with 13 years of follow-up found different effects on depression for mentally passive and mentally active sedentary behaviors^26^, with a significant beneficial influence of mentally active sedentary behaviors on incident depression and non-significant effect of mentally passive sedentary behaviors or total sedentary behaviors on the risk of depression.

Epidemiological evidence on the association between sedentary behaviors and risk of depression are inconsistent^23,25–28^. It remains unclear whether there is a relation between sedentary behaviors and the risk of depression in adults or not? Pooling existing evidence, we conducted a meta-analysis of prospective studies to examine the association of sedentary behaviors with the risk of depression.

## Methods

We designed and conducted this review under the guidelines of the Meta-analyses Of Observational Studies in Epidemiology (MOOSE)^29^.

### Search strategy

We systematically searched the electronic databases of PubMed and Embase through June 2019 to identify studies examining the relationship between sedentary behaviors and the risk of depression. The following terms and their combinations were used in the search: “watching television”, “screen time”, “sitting”, “computer use”, “media use”, “car driving”, “sedentary time”, “sedentary behaviors”, and “depression”. We also reviewed the references of included articles to identify any eligible studies omitted by previous database searches.

### Inclusion criteria

Studies meeting the following criteria were included in this meta-analysis: (1) the study design was prospective; (2) the exposure was sedentary behaviors, including watching television, using a computer, reading, driving, and so on; (3) the outcome was depression; (4) the study population was average adults; (5) the study reported relative risk (RR) or hazard ratio (HR), with their 95% confidence intervals (CIs) available or accountable.

### Data extraction

For all included studies, we extracted information on the year of publication, name of the first author, country, follow-up year, follow-up rate, number of participants, average age of participants at baseline, depression diagnosis measure, cohort name, specified sedentary behaviors, RR with their 95% CIs, and adjusted variables. All the extracted RRs were adjusted for most covariates.

### Quality assessment

The quality of included studies were assessed by the nine-point Newcastle-Ottawa scale (NOS)^30^, which is a common tool in meta-analysis for observational studies. The scale awards prospective research a maximum of nine points (four for selection quality, two for comparability, and three for outcome of interests). A total score of 0–3 was considered as low quality, 4–6 was considered as moderate quality, and 7–9 was assigned to high quality.

### Statistical analyses

We primarily used the full-adjusted RRs to pool results, except for one study with crude RR^31^. For studies where sedentary time was divided into several levels, RRs were recalculated by pooling values of upper groups, whereas the lowest level of sedentary time was taken as reference. Only one study took the highest classification as reference group, so we used a reciprocal function to process original data^32^.

We conducted subgroup analyses and meta-regression analyses to explore potential heterogeneity across studies. Stratified analyses were performed, respectively, by covariates, specific sedentary behavior, depression diagnosis criteria, and baseline depression status (excluded or adjusted). As there was currently no clear definition for mentally passive and mentally active sedentary behaviors, we referred to one previous study that it took sedentary behaviors including watching television, chatting while sitting and sitting around as “mentally passive”, whereas interpreted using a computer, reading, and car driving as “mentally active”^3^. According to the NOS degree, we specifically excluded low quality studies to examine whether the primarily pooled result was stable. Simultaneously, sensitivity analyses were performed by leaving-one-out procedure to evaluate the underlying between-study heterogeneity.

Statistical heterogeneity across studies was estimated by *I*^2^ of Higgins and Thompson^33^, with values of 25%, 50%, and 75%, representing heterogeneity levels of low, moderate, and high, respectively. The DerSimonian and Laird random-effects model was used to synthesize the RRs when a relatively high heterogeneity was detected, otherwise, the fixed-effects model was used.

Potential publication bias was visually inspected through funnel plot by referencing the Begg correlation test and Egger linear regression test^34,35^. All the statistical analyses were performed with STATA V.12.0 (StataCorp, College Station, Texas, USA), with a two-sided significance level of 0.05.

## Results

### Literature selection

**Fig. 1:**
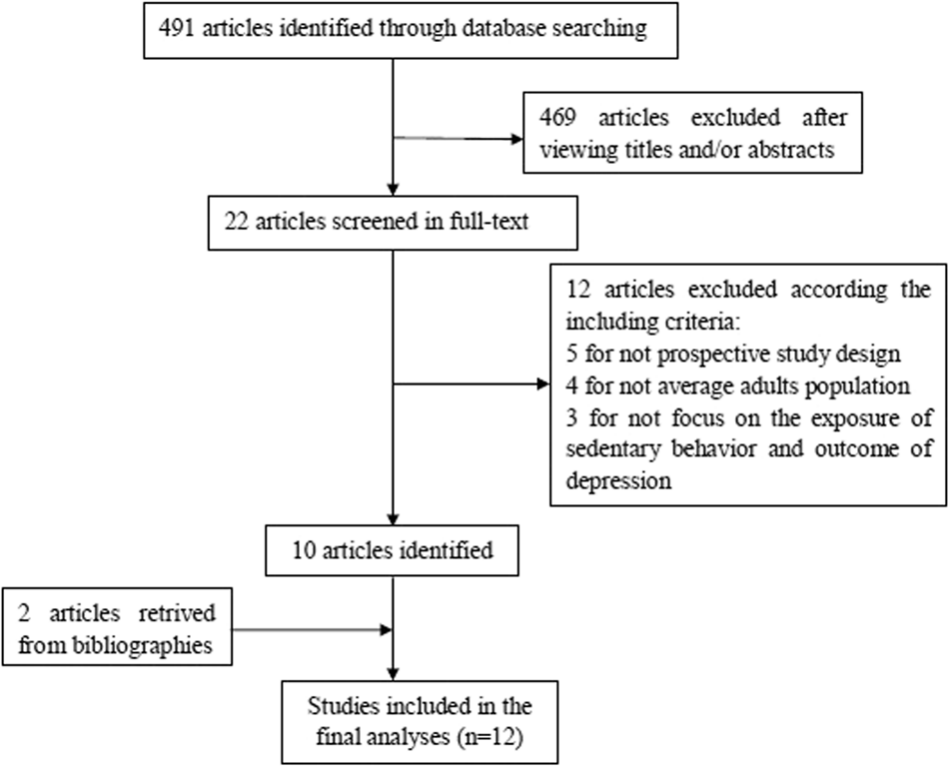
Flow chart of study identification.

A total of 491 relevant articles were found by searching the databases, of which 469 articles were precluded after reviewing titles and/or abstracts. Subsequently, 12 articles were excluded after full-text review for not complying with the inclusion criteria, including one that focused on the association in post-pregnant women. Another two studies were further identified from the references of qualified articles. A total of 12 literatures^25–28,31,32,36–41^ were finally included in this meta-analysis. The study selection procedure is presented in the flow chart in Fig. 1.

### Study characteristics

The characteristics of included studies are presented in Table 1. Among them, two studies were conducted in the United States^25,37^, three were in Australia^26,38,40^, and the other seven studies were performed in Europe, including Finland, Sweden, Denmark, Spain, and the United Kingdom^27,28,31,32,36,39,41^. The follow-up time ranged from 1 to 13 years, with an average of 5.97 years. All included studies had high follow-up rates of above 90%. The total number of participants was 128,553. Eight studies excluded the baseline cases of depression in their cohort design^25,28,31,36–39,41^, three studies adjusted for the baseline depression status in their analyses^26,27,40^, and one study neither excluded nor adjusted for the baseline depression status^32^. With regard to the criteria of depression diagnosis, one study did not report any criteria^38^, four studies used the CES-D-10 (versions of the Centre for Epidemiological Studies Depression Scale) criteria^25,27,32,40^, and the other seven studies used different criteria including the Finnish modified version of Beck’s 3-item depression scale^36^, the Primary Care Evaluation of Mental Disorders screening form^31,41^, the Major Depression Inventory^28^, physician diagnosis^37,39^, and medical registers^26^. All of the studies adjusted for multiple variables, including basic demographic characteristics, smoking status, body mass index (BMI), alcohol consumption, physical activity, and so on, except for one with unadjusted results^31^. Classification of sedentary behaviors differed among studies, four of which reported total screen time^36–39^, whereas the others classified sedentary behaviors as computer use^25,27,28,38,40^, watching television^25,27,28,32,37,40^, being a passenger or driver in a car^25^, and so on. Two studies explored the effects in women only^38,40^, three studies reported the RRs in men and women, respectively^27,31,41^, and the other included studies did not classify by gender^25,26,28,32,36,37,39^.

**Table 1:** Characteristics of studies included in the meta-analysis.

First author	Year	Country	Follow-up time (y)	Follow-up rate (%)	*N*	Age at baseline	Depression at baseline	Depression diagnosis	SBs category	RR (95%CI)	NOS score	Covariates adjustment
Lampinen^36^	2003	Finland	8	90	384	72.4	Excluded	RBDI	–	1.01 (0.46, 2.21)	9	Gender, age, chronic illnesses, and length of education
Thomee^31^	2007	Sweden	1	94	1127	21.5	Excluded	Prime-MD screening	CU, phone, chat, e-mail	1.13 (0.91, 1.40)	5	–
Sanchez-villegas^39^	2008	Spain	6	90	10381	43.1	Excluded	Physician diagnosis	–	1.12 (0.99, 1.28)	8	Age, sex, energy intake, smoking, marital status, arthritis, ulcer, cancer
Lucas^37^	2011	USA	10	91	49821	42.5	Excluded	Physician diagnosis	TW	1.07 (1.01, 1.14)	8	Age, time interval, BMI, TV category, marital status, social relation, smoking status, energy intake, coffee, diabetes, hypertension
Thomee^41^	2012	Sweden	1	92	4163	22	Excluded	Prime-MD screening	CU, chat, e-mail, Computer game	1.12 (0.80, 1.57)	8	Socio-demographic, education, relationship status, occupation
Peeters^38^	2013	Australia	3	93	9452	55	Excluded	NR	–	1.03 (0.88, 1.21)	7	Age, area of residence, education, PA, BMI, smoking status, alcohol
Hamer^32^	2014	UK	2	94	6359	64.9	Unadjusted	CES-D-8	TW, CU, print media	1.18 (1.11, 1.25)	7	Age, sex, smoking, PA, alcohol, social class, BMI, disability, chronic illness
Teychenne^40^	2014	Australia	3	95	1511	31.5	Adjusted	CES-D-10	TW,CU	0.84 (0.55, 1.29)	7	Age, education, children living at home, employment, PA, marital status, BMI
Grontved^28^	2015	Denmark	12	96	435	16	Excluded	MDI	TW, CU	1.57 (1.16, 2.12)	9	Age, follow-up time, sex, smoking, parental education level, alcohol, parental marital status, school id
Sui^25^	2015	USA	9.3	97	4802	49	Excluded	CES-D-10	TW, car riding	1.54 (1.20, 1.97)	8	Age, gender, education, BMI, marital status, smoking, employment, diabetes
Andrade-Gomez^27^	2018	Spain	3.3	98	2614	60	Adjusted	CES-D-10	TW, CU	0.96 (0.83, 1.12)	8	Age, educational level, baseline GDS scores, PA, smoking, chronic disease, cancer
Hallgren^26^	2018	Australia	13	99	37504	51.5	Adjusted	Medical registers	Mentally passive, mentally active	0.91 (0.75, 1.10)	9	Age, gender, BMI, employment status, education, MVPA

*RBDI* the Finnish modified version of Beck’s 13-item depression scale, *Prime-MD screening* the Primary Care Evaluation of Mental Disorders screening form, *CES-D* versions of the Centre for Epidemiological Studies Depression Scale, *MDI* the Major Depression Inventory, *NR* not report, *TW* television watching, *CU* computer using, *BMI* body mass index, *GDS score* score counted by the10-item version of the Geriatric Depression Scale (GDS-10), *PA* physical activity, *MVPA* moderate-to-vigorous physical activity

The average NOS score was 7.75. All the included studies, except for one with the score equal to 5^31^, were defined as high quality (Table 2).

**Table 2:** Methodological quality of included cohort studies based on the Newcastle-Ottawa Scale.

Study ID	Selection				Comparability of factors control	Outcome			Total score
	Representative of the exposed	Selection of the non-exposed	Ascertainment of exposure	Outcome of interest not presented at start		Outcome assessment	Long enough follow-up	Adequacy of follow-up	
Lampinen, 2003^36^	✩	✩	✩	☆	☆☆	☆	☆	☆	9
Thomee, 2007^31^	–	✩	✩	–	–	☆	–	☆	5
Sanchez-villegas, 2008^39^	–	✩	✩	☆	☆☆	☆	☆	☆	8
Lucas, 2011^37^	–	✩	✩	☆	☆☆	☆	☆	☆	8
Thomee, 2012^41^	☆	✩	✩	–	☆☆	☆	☆	☆	8
Peeters, 2013^38^	☆	✩	✩	–	☆☆	–	☆	☆	7
Hamer, 2014^32^	☆	✩	✩	–	☆☆	☆	–	☆	7
Teychenne, 2014^40^	–	✩	✩	–	☆☆	☆	☆	☆	7
Grontved, 2015^28^	☆	✩	✩	☆	☆☆	☆	☆	☆	9
Sui, 2015^25^	–	✩	✩	☆	☆☆	☆	☆	☆	8
Andrade-Gomez, 2018^27^	☆	☆	☆	–	☆☆	☆	☆	☆	8
Hallgren, 2018^26^	☆	☆	☆	☆	☆☆	☆	☆	☆	9

### Association between sedentary behavior and the risk of depression

**Fig. 2:**
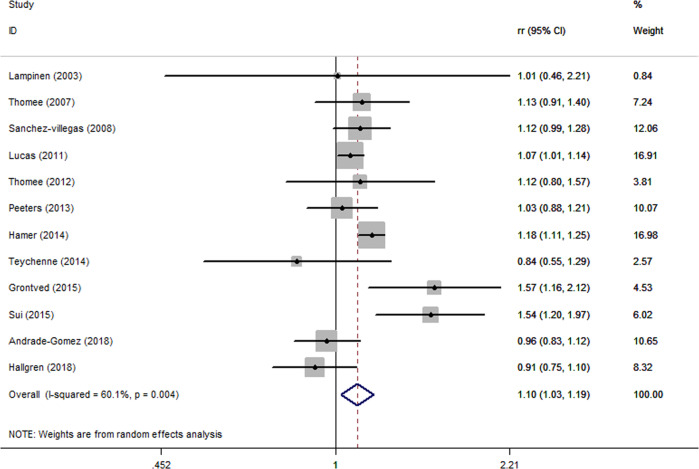
Forest plot of sedentary behavior associated with depression.

Figure 2 shows the multivariable-adjusted RRs of included studies, as well as the pooled result of the random-effect model. The pooled RR was 1.10 (95% CI: 1.03,1.19), which showed that sedentary behaviors were positively associated with risk of depression, with a moderate heterogeneity (*P* = 0.003; *I*^2^ = 60.6%).

### Subgroup analyses

When stratified by specific sedentary behavior, we found that watching television was positively associated with the risk of depression (RR = 1.18, 95% CI 1.07–1.30), whereas using a computer was statistically non-significant (RR = 0.99, 95% CI 0.79–1.23). Mentally passive sedentary behaviors could increase the risk of depression (RR = 1.17, 95% CI 1.08–1.27), whereas the effect of mentally active sedentary behaviors were non-significant (RR = 0.98, 95% CI 0.83–1.15). We observed non-significant pooled effects after stratification by gender, which was consistent with original studies^27,31,38,40,41^. Sedentary behaviors were positively related to depression defined by clinical diagnosis (RR = 1.08, 95% CI 1.03, 1.14), whereas the association was statistically non-significant when depression was evaluated by the CES-D (RR = 1.12, 95% CI 0.94,1.34), Prime-MD screening (RR = 1.08, 95% CI 1.01–1.17) and other criteria such as the Major Depression Inventory and medical registers. The pooled result of studies that excluded baseline depression in their cohort or analyses suggested a significantly positive association between sedentary behavior and risk of depression, whereas the summarized effect of studies that adjusted for baseline depression in their analyses showed a non-significant negative relation with the risk of depression. Results having adjusted for smoking, as well as, not adjusting for BMI and physical activity showed a positive association on this issue, as shown in Table 3. All the meta-regression analyses showed non-significant interaction effects, which weakened our explanation for the possible source of assumed heterogeneity. Whether adjusted for covariates or not, values of *I*^2^ fluctuated substantially. Compared with the pooled result of unadjusted studies’, *I*^2^ changed from 23.1% to 73% when we pooled the results of studies which adjusted for BMI. (Table 3).

**Table 3:** Subgroup analyses of sedentary behavior and depression risk.

Stratification group	Number of studies	Relative risk	95% confidence interval	Heterogeneity test			
				*Q*	*I*^2^ (%)	*P* value	*P* for interaction
*Sedentary behavior*							
Using a computer	5	1.01	(0.86, 1.17)	11.45	56.3	0.043	0.326
Watching television	6	1.18	(1.07, 1.30)	17.89	72.0	0.003	
Mentally active	7	0.98	(0.83, 1.15)	17.59	65.9	0.007	0.315
Mentally passive	8	1.17	(1.08, 1.27)	17.95	61.0	0.012	
*Sex*							
Men	3	0.97	(0.85, 1.11)	1.76	0.00	0.414	0.197
Women	5	1.14	(0.96, 1.34)	2.57	0.00	0.632	
Combined	7	1.15	(1.05, 1.27)	21.19	71.7	0.002	
*Depression definition*							
Physician diagnosis	2	1.08	(1.03, 1.14)	0.43	0.00	0.513	0.740
Prime-MD screening	2	0.98	(0.81, 1.20)	0.25	0.00	0.616	
CES-D criteria	4	1.12	(0.94, 1.34)	13.13	77.2	0.004	
Other criteria	4	1.09	(0.85, 1.40)	10.92	72.5	0.012	
*Baseline depression*							
Excluded	8	1.15	(1.05, 1.27)	14.07	50.2	0.050	0.665
Adjusted	3	0.93	(0.83, 1.05)	0.44	0.00	0.803	
*Covariates adjusted*							
Smoking							
Adjusted	6	1.12	(1.05, 1.20)	12.73	60.7	0.026	0.897
Not adjusted	6	1.10	(0.90, 1.33)	12.48	59.9	0.029	
Body mass index							
Adjusted	6	1.06	(0.93, 1.22)	21.97	77.2	0.001	0.470
Not adjusted	6	1.12	(1.03, 1.21)	6.50	23.1	0.261	
Alcohol							
Adjusted	4	1.13	(0.97, 1.31)	12.40	75.8	0.006	0.812
Not adjusted	8	1.10	(1.00, 1.21)	13.10	46.5	0.070	
Physical activity							
Adjusted	4	1.03	(0.90, 1.18)	12.27	75.5	0.007	0.195
Not adjusted	8	1.17	(1.05, 1.30)	15.17	53.8	0.034	

*I*^2^ is interpreted as the proportion of total variation across studies that are owing to heterogeneity rather than chance

*Prime-MD screening* the Primary Care Evaluation of Mental Disorders screening form, *CES-D* versions of the Centre for Epidemiological Studies Depression Scale

### Sensitivity analyses

Sensitivity analysis was performed by omitting one study in turn, with the pooled RRs fluctuating between 1.08 (95% CI 1.01, 1.16) and 1.12 (95% CI 1.04, 1.21), which supported the stability of our results. In addition, the RR was 1.10 (95% CI 1.02, 1.19) after removing the low-quality study^31^, which further supported that sedentary behavior was related to depression.

### Publication bias

**Fig. 3:**
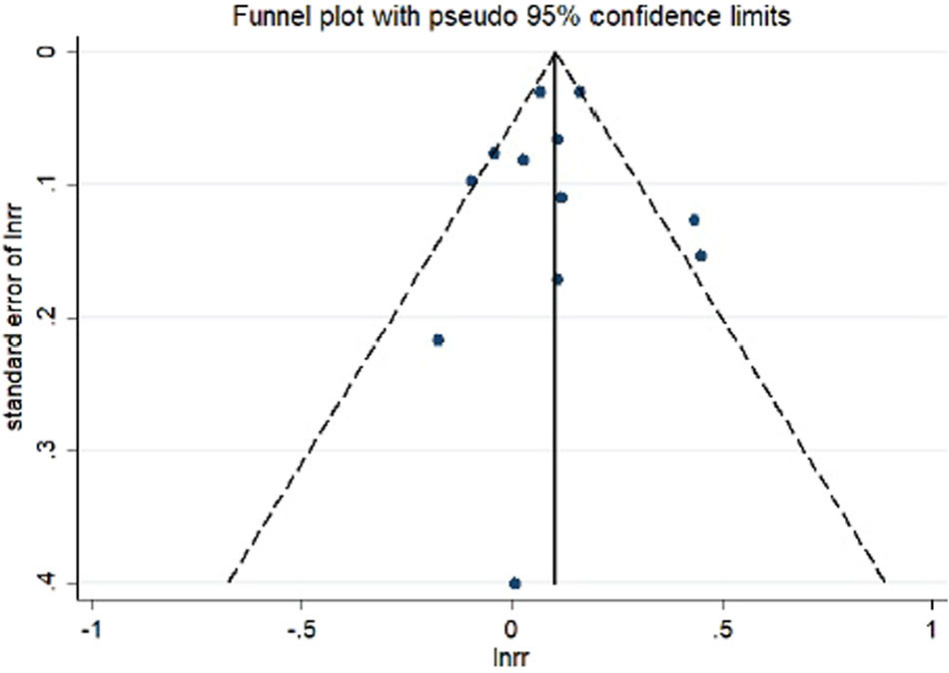
Funnel plot for studies of sedentary behavior and depression. The horizontal line represents summary effect estimates, and the dotted lines are pseudo 95% CIs.

Evidence of publication bias was visually inspected through the funnel plot (Fig. 3) and neither the Egger’s test nor the Begg’s test indicated statistically significant potential for publication bias in either case (Egger, *P* = 0.939; Begg, *P* = 1.000).

## Discussion

In this meta-analysis based on 12 prospective cohort studies, we found that sedentary behaviors, especially mentally passive behaviors, were positively associated with the risk of depression. Each additional increment of time spent on passively sedentarily watching television could increase the risk of depression, but the estimated effects differed by covariates adjustment. The pooled results stratified by gender were non-insignificant. Subgroup analyses suggested that sedentary behavior was positively related to depression defined by physician diagnosis, whereas the pooled effects were statistically non-significant when depression was diagnosed by other criteria.

There are mechanisms explaining the association between sedentary behaviors and risk of depression. First, sedentary behaviors like using a computer can hinder direct communication between individuals, causing a reduction in social interaction and increasing the potential for depression^42^. Second, sedentary behaviors also cut back on time spent in physical exercise, which is an effective prevention and treatment for depression^43–45^. Many observational studies have focused on the association between sedentary behaviors and the risk of depression, whereas evidence from experimental studies is still limited. Experimental studies investigating the biological mechanisms of this association are still needed to provide more powerful explanations.

Interestingly, we found that mentally active sedentary behaviors, as well as using a computer, were unrelated to the risk of depression (*P* > 0.05). As mentioned above, using a computer is one kind of mentally positive sedentary behaviors, which could be beneficial to mental health^3^. A previous prospective cohort study showed that there were protective effect of mentally active sedentary behaviors on the incidence of depression^26^. However, original studies that took computer use as the exposure were more likely to find null or negative relationship between sedentary behaviors and the risk of depression^46^. What’s more, a previous meta-analysis suggested that computer use could increase the risk of depression^23^. As the findings are contradictory, future studies focusing on the relationship between using a computer or mentally active sedentary behaviors and risk of depression with its underlying mechanisms should be encouraged. Also, as we found that mentally passive sedentary behaviors, such as watching television, could be detrimental to mental health and may increase the risk of depression, the distinct association between mentally passive and mentally active sedentary behaviors with their effects on depression should also be brought into attention.

Subgroup analysis also showed that sedentary behavior was detrimental for the risk of depression when unadjusted for physical activity. Studies focusing on the combined effect of physical activity and sedentary behaviors on depression have reported inconsistent conclusions. Two observational studies found that people with more time spent in physical activity and less time spent in sedentary behaviors were more likely to develop depression^47,48^. Also, the substitution of sedentary behaviors with physical activity was found to be protective for depression^4^. However, a randomized controlled trial did not observe the joint effect of sedentary behaviors and exercise on depression^49^. As sedentary behavior and physical activity are both common human activities and generally exist in our daily life, future studies are needed to determine whether or which combinations of the two behaviors could protect against depression.

Most of the subgroup effects were statistically non-significant, which might be attributed to methodological discrepancies between studies. For all included studies, the shortest follow-up time was 1 year, which might not be long enough for the occurrence of depression. The difference of sample sizes might influence the statistical power of original studies. In addition, the elderly and new adults differed in the prevalence of depression, which contributed to the heterogeneity between studies, and thus influenced the results in our subgroup analyses. The number of original studies classified by gender was limited, which might be the cause of non-significant results in stratification analyses by sex. As mentioned before, diagnoses criteria of depression seemed to influence the results, which suggested that different criteria might affect the sensitivity and specificity of depression diagnoses. Prospective cohort studies starting from exposures and observing the occurrence of outcome provided stronger evidence for a cause–effect association than other observational studies^50^. Among included studies in this meta-analysis, those with depression cases pre-excluded have stronger statistical power than those with baseline depression adjusted in their statistical models. Considering the weakening effects caused by adjustment of baseline depression in statistical analyses of original studies and the limitation in the number of studies, it is not surprising that the pooled effect in this subgroup was non-significant. Statistical heterogeneity might be related to the differences in adjustment factors. Non-adjustment for drinking and physical activity contributed to the positive association between sedentary behaviors and depression, as alcohol and lacking physical activity are both independent risk factors for depression^45,51^.

The observed heterogeneity was mostly significant in subgroup analyses. *I*^2^ fluctuated especially in groups of stratification by gender, ways of diagnosing depression, whether baseline depression cases were excluded in the analyses and whether BMI was adjusted for. But all the meta-regression analyses showed non-significant interaction effects, which weakened the possibility of heterogeneity caused by these three factors. After excluding one low-quality study^31^, the *I*^2^ of pooled value did not change materially, thus eliminating the possibility that study quality influences between-study heterogeneity. Future studies considering the disturbing factors above are needed to determine the underlying heterogeneity.

Not only is sedentary behavior a risk factor for depression, studies have also suggested that depression may have an effect on sedentary status. Studies focused on sedentary status among depression patients have presented that adults with depression engaged in low levels of physical activity and high levels of sedentary behaviors^52,53^. In addition, for adolescents, evidence also suggested that sedentary behaviors increase the risk of depression^24,54^. As sedentary behaviors and depression have interaction in different age groups, slightly restricting sedentary behaviors may produce great benefits for human health.

Strengths of this meta-analysis should be mentioned. First, the studies included in our analyses were all prospective, which can provide strong reliability of results. Second, except for one low-quality study that did not report adjusted RR, all the original studies were of high quality and reported multivariate adjusted RRs. Third, our study was based on adults and had a large number of participants, with which we can provide powerful statistical evidence for the estimated effects. Fourth, this is the first meta-analysis that classifies sedentary behaviors into mentally active and mentally passive, and discusses their effects on depression respectively.

Limitations should also be acknowledged. First, owing to limited information on original studies, we did not conduct dose–response analyses and were unable to explore the relationship between other forms of sedentary behaviors (like online chatting and car driving) and risk of depression. Second, we did not find out credible evidence for sources of heterogeneity, which were needed to be supported by further research. Third, this meta-analysis did not deal with possible existing bias caused by differences in methodology between studies. Fourth, we simply took computer use as mentally active sedentary behavior, whereas it could also be mentally passive when a computer was used for watching television, film, etc. As there was no clear definition for the two kinds of sedentary behaviors, future studies could pay attention to developing questionnaires that accurately measure these diverse behaviors and building up a classification system.

In conclusion, our analyses suggest there is a positive association between mentally passive sedentary behavior and risk of depression, and the increment of time spent on sedentarily watching television could increase the risk of depression. Given the increasing prevalence of depression and widespread sedentary behaviors in modern society, the results of our study are of importance for clinical and public health. Restrictions on mentally passive sedentary behaviors should be recommended to prevent depression. As depression is just one kind of mental disorder, future researches focusing on the impact of interruptions in sedentary behaviors on other aspects of mental health also should be brought into attention.

## References

[1] McCarthy M (2017). Fitness moderates glycemic responses to sitting and light activity breaks. Med. Sci. Sports Exerc..

[2] Sedentary Behaviour Research N. (2012). Letter to the editor: standardized use of the terms” sedentary” and” sedentary behaviours”. Appl. Physiol. Nutr. Metab..

[3] Kikuchi H (2014). Distinct associations of different sedentary behaviors with health-related attributes among older adults. Prev. Med..

[4] Hallgren M., et al. Cross-sectional and prospective relationships of passive and mentally active sedentary behaviours and physical activity with depression. *Br. J. Psychiatry* **21**, 1–7 (2019). 10.1192/bjp.2019.60 30895922

[5] Carter S, Hartman Y, Holder S, Thijssen DH, Hopkins ND (2017). Sedentary behavior and cardiovascular disease risk: mediating mechanisms. Exerc. sport Sci. Rev..

[6] Gao Y (2017). Effects of sedentary occupations on type 2 diabetes and hypertension in different ethnic groups in North West China. Diabetes Vasc. Dis. Res..

[7] Hamilton MT, Hamilton DG, Zderic TW (2014). Sedentary behavior as a mediator of type 2 diabetes. Med. Sport Sci..

[8] Lockyer M (2016). Sedentary behaviour associated with type 2 diabetes. Practitioner.

[9] Pandey A (2016). Continuous dose-response association between sedentary time and risk for cardiovascular disease: a meta-analysis. JAMA Cardiol..

[10] Biswas A (2015). Sedentary time and its association with risk for disease incidence, mortality, and hospitalization in adults: a systematic review and meta-analysis. Ann. Intern. Med..

[11] An KO, Jang JY, Kim J (2015). Sedentary behavior and sleep duration are associated with both stress symptoms and suicidal thoughts in Korean adults. Tohoku J. Exp. Med..

[12] Ashdown-Franks G (2018). Sedentary behavior and perceived stress among adults aged >/=50 years in six low- and middle-income countries. Maturitas.

[13] Falck RS, Davis JC, Liu-Ambrose T (2017). What is the association between sedentary behaviour and cognitive function? A systematic review. Br. J. Sports Med..

[14] Yang Y, Shin JC, Li D, An R (2017). Sedentary behavior and sleep problems: a systematic review and meta-analysis. Int. J. Behav. Med..

[15] Gelenberg AJ (2010). The prevalence and impact of depression. J. Clin. Psychiatry.

[16] Smith K (2014). Mental health: a world of depression. Nature.

[17] Alexopoulos GS. Depression in the elderly. **365**, 1961–1970 (2005). 10.1016/S0140-6736(05)66665-2 15936426

[18] Sibeoni J. F., Moro M. R. Childhood and adolescent depression. **75**, 73–80 (2014). 24855783

[19] Jorm AF (1987). Sex and age differences in depression: a quantitative synthesis of published research. Aust. N. Z. J. Psychiatry.

[20] Salk RH, Hyde JS, Abramson LY (2017). Gender differences in depression in representative national samples: meta-analyses of diagnoses and symptoms. Psychol. Bull..

[21] Schuch JJ, Roest AM, Nolen WA, Penninx BW, de Jonge P (2014). Gender differences in major depressive disorder: results from the Netherlands study of depression and anxiety. J. Affect. Disord..

[22] Mannan M, Mamun A, Doi S, Clavarino A (2016). Prospective associations between depression and obesity for adolescent males and females- a systematic review and meta-analysis of longitudinal studies. PLoS ONE.

[23] Zhai L, Zhang Y, Zhang D (2015). Sedentary behaviour and the risk of depression: a meta-analysis. Br. J. Sports Med..

[24] Liu M, Wu L, Yao S (2016). Dose-response association of screen time-based sedentary behaviour in children and adolescents and depression: a meta-analysis of observational studies. Br. J. Sports Med..

[25] Sui X (2015). Associations between television watching and car riding behaviors and development of depressive symptoms: a prospective study. Mayo Clin. Proc..

[26] Hallgren M (2018). Passive and mentally-active sedentary behaviors and incident major depressive disorder: a 13-year cohort study. J. Affect. Disord..

[27] Andrade-Gomez E, Martinez-Gomez D, Rodriguez-Artalejo F, Garcia-Esquinas E (2018). Sedentary behaviors, physical activity, and changes in depression and psychological distress symptoms in older adults. Depress. Anxiety.

[28] Grontved A (2015). A prospective study of screen time in adolescence and depression symptoms in young adulthood. Prev. Med..

[29] Stroup DF (2000). Meta-analysis of observational studies in epidemiology: a proposal for reporting. Meta-analysis Of Observational Studies in Epidemiology (MOOSE) group. JAMA.

[30] Wells G., et al. The Newcastle-Ottawa Scale (NOS) for assessing thequality of nonrandomised studies in meta-analyses. Available at: http://wwwohrica/programs/clinical_epidemiology/oxfordasp (2012).

[31] Thomee S, Eklof M, Gustafsson E, Nilsson R, Hagberg M (2007). Prevalence of perceived stress, symptoms of depression and sleep disturbances in relation to information and communication technology (ICT) use among young adults - an explorative prospective study. Computers Hum. Behav..

[32] Hamer M, Stamatakis E (2014). Prospective study of sedentary behavior, risk of depression, and cognitive impairment. Med. Sci. sports Exerc..

[33] Higgins JP, Thompson SG, Deeks JJ, Altman DG (2003). Measuring inconsistency in meta-analyses. BMJ.

[34] Begg CB, Mazumdar M (1994). Operating characteristics of a rank correlation test for publication bias. Biometrics.

[35] Egger M, Davey Smith G, Schneider M, Minder C (1997). Bias in meta-analysis detected by a simple, graphical test. BMJ.

[36] Lampinen P, Heikkinen E (2003). Reduced mobility and physical activity as predictors of depressive symptoms among community-dwelling older adults: an eight-year follow-up study. Aging Clin. Exp. Res..

[37] Lucas M (2011). Relation between clinical depression risk and physical activity and time spent watching television in older women: a 10-year prospective follow-up study. Am. J. Epidemiol..

[38] Peeters GMEE, Burton NW, Brown WJ (2013). Associations between sitting time and a range of symptoms in mid-age women. Prev. Med..

[39] Sanchez-Villegas A (2008). Physical activity, sedentary index, and mental disorders in the SUN Cohort Study. Med. Sci. Sports Exerc..

[40] Teychenne M, Abbott G, Ball K, Salmon J (2014). Prospective associations between sedentary behaviour and risk of depression in socio-economically disadvantaged women. Prev. Med..

[41] Thomee S., Harenstam A., Hagberg M. Computer use and stress, sleep disturbances, and symptoms of depression among young adults - a prospective cohort study. *BMC Psychiatry* **12**, 176 (2012). 10.1186/1471-244X-12-176 PMC3528646 23088719

[42] Kraut R (1998). Internet paradox. A social technology that reduces social involvement and psychological well-being?. Am. Psychol..

[43] Biddle SJ, Asare M (2011). Physical activity and mental health in children and adolescents: a review of reviews. Br. J. Sports Med..

[44] Gujral S, Aizenstein H, Reynolds CF, Butters MA, Erickson KI (2017). Exercise effects on depression: possible neural mechanisms. Gen. Hosp. Psychiatry.

[45] Lubans, D. et al. Physical activity for cognitive and mental health in youth: a systematic review of mechanisms. *Pediatrics* 2016; **138**, pii: e20161642. 10.1542/peds.2016-1642 27542849

[46] Teychenne M., Ball K Fau, Salmon J. Sedentary behavior and depression among adults: a review. *Int. J. Behav. Med.* **17**, 246–254 (2010). 10.1007/s12529-010-9075-z 20174982

[47] Santos D. A., Virtuoso JS Jr, Meneguci J., Sasaki J. E., Tribess S. Combined associations of physical activity and sedentary behavior with depressive symptoms in older adults. *Issues Ment. Health Nurs.* **38**, 272–276 (2017). 10.1080/01612840.2016.1263695 28287869

[48] Liao Y., Shibata A., Ishii K., Oka K. Independent and combined associations of physical activity and sedentary behavior. *Int. J. Behav. Med.* **23**, 402–409 (2016). 10.1007/s12529-015-9484-0 25850784

[49] Blough J., Loprinzi P. D. Experimentally investigating the joint effects of physical activity and sedentary behavior on depression and anxiety: a randomized controlled trial. *J. Affect. Disord.* **239**, 258–268 (2018). 10.1016/j.jad.2018.07.019 30029153

[50] Shen Y, Zhang S, Zhou J, Chen J (2017). Cohort research in “Omics” and preventive medicine. Adv. Exp. Med. Biol..

[51] Boden J. M., Fergusson D. M. Alcohol and depression. *Addiction*. **106**, 906–914 (2011). 10.1111/j.1360-0443.2010.03351.x 21382111

[52] Schuch F (2017). Physical activity and sedentary behavior in people with major depressive disorder: a systematic review and meta-analysis. J. Affect. Disord..

[53] Vancampfort D (2017). Sedentary behavior and physical activity levels in people with schizophrenia, bipolar disorder and major depressive disorder: a global systematic review and meta-analysis. World Psychiatry.

[54] Vancampfort D, Stubbs B, Firth J, Van Damme T, Koyanagi A (2018). Sedentary behavior and depressive symptoms among 67,077 adolescents aged 12-15 years from 30 low- and middle-income countries. Int. J. Behav. Nutr. Phys. Act..

